# Ventilatory response to head‐down‐tilt in healthy human subjects

**DOI:** 10.1113/EP092014

**Published:** 2024-10-24

**Authors:** Abdulaziz Alsharifi, Niamh Carter, Akbar Irampaye, Charlotte Stevens, Elisa Mejia, Joerg Steier, Gerrard F. Rafferty

**Affiliations:** ^1^ Centre for Human and Applied Physiological Sciences (CHAPS), Faculty of Life Sciences and Medicine King's College London London UK; ^2^ Department of Respiratory Therapy, College of Applied Medical Sciences Jazan University Jazan Saudi Arabia; ^3^ Lane Fox Unit/Sleep Disorders Centre Guy's & St Thomas’ NHS Foundation Trust London UK

**Keywords:** baroreceptors, cerebral blood flow, chemoreceptors, head‐down tilt, respiratory control, Trendelenburg position, ventilation

## Abstract

Postural fluid shifts may directly affect respiratory control via a complex interaction of baro‐ and chemo‐reflexes, and cerebral blood flow. Few data exist concerning the steady state ventilatory responses during head‐down tilt. We examined the cardiorespiratory responses during acute 50° head‐down tilt (HDT) in 18 healthy subjects (mean [SD] age 27 [10] years). Protocol 1 (*n* = 8, two female) was 50° HDT from 60° head‐up posture sustained for 10 min, while exposed to normoxia, normoxic hypercapnia (5% CO_2_), hypoxia (12% inspired O_2_) or hyperoxic hypercapnia (95% O_2_, 5% CO_2_). Protocol 2 (*n* = 10, four female) was 50° HDT from supine, sustained for 10 min, while breathing either medical air or normoxic hypercapnic (5% CO_2_) gas. Ventilation (${\dot{V}}_{E}$, pneumotachograph), end‐tidal O_2_ and CO_2_ concentration and blood pressure (Finapres) were measured continuously throughout each protocol. Middle cerebral artery blood flow velocity (MCAv; transcranial Doppler) was also measured during protocol 2. Ventilation increased significantly (*P *< 0.05) compared to baseline during HDT in both hyperoxic hypercapnia (protocol 1 by mean [SD] 139 [26]%) and normoxic hypercapnia (protocol 1 by mean [SD] 131 [21]% and protocol 2 by 129 [23]%), despite no change in $P_{\mathrm{ETC}O_{2}}$ or $P_{\mathrm{ET}O_{2}}$ from baseline. No change in ${\dot{V}}_{E}$ was observed during HDT with medical air or hypoxia, and there was no significant change in MCAv during HDT compared to baseline. The absence of change in cerebral blood flow leads us to postulate that the augmented ventilatory response during steep HDT may involve mechanisms related to cerebral venous pressure and venous outflow.

## Abstract (highlights)

- **What is the central question of this study?**
  Postural fluid shifts affect respiratory control via complex interactions between baroreceptor and chemoreceptor reflexes, and cerebral blood flow. Few data exist concerning steady state ventilatory responses during head down tilt.
- **What is the main finding and its importance?**
  Acute 50° head‐down tilt augments the ventilatory response to steady state normoxic and hyperoxic hypercapnia possibly via mechanisms involving cerebral blood flow. These findings are relevant clinically during procedures performed in a steep head‐down position and microgravity environments where individuals are exposed to fluid shifts and elevated atmospheric CO_2_.

## 1 INTRODUCTION

Head‐down tilting (HDT) is used in research to simulate the effects of microgravity in space analogue studies (Lawley et al., 2017; Watenpaugh, 2016) and clinically where steep head‐down tilt (steep Trendelenburg position 30–45° HDT) is used in surgery, and results in a shift of up to 1 L of blood from the lower extremities to the central circulation (Martin, 1995). The fluid shifts that occur result in multiple cardiovascular and cerebrovascular adaptations (Bundgaard‐Nielsen et al., 2009; Marshall‐Goebel et al., 2016; Pavy‐Le Traon et al., 2007; Willie et al., 2014). Increased thoracic blood volume also leads to decreased lung elastic recoil and compliance, and increased airway resistance (Prisk, 2000). The change in the gravitational vector also results in conformational changes in the lungs, diaphragm, abdominal contents and chest wall affecting lung volumes and respiratory pattern (Estenne et al., 1992; Prisk, 2000; Steier et al., 2009) as well as alterations in pulmonary blood flow distribution (Henderson et al., 2006). Despite such recognised effects of head‐down tilt on the lung and chest wall alongside the cardiovascular and cerebrovascular control, there are few data describing the effects of head‐down postures on respiratory control.

Postural fluid shifts may impact on respiratory control via baroreflex, peripheral and central chemoreflex, and cerebral blood flow‐mediated mechanisms. Reduced ventilatory responses to chemoreceptor stimulation during hypertension have been observed in dogs (Heistad et al., 1974; Hopp & Seagard, 1998) and Stewart et al. (2011) demonstrated in humans that rapid baroreflex unloading was a potent ventilatory stimulus. While increases in the hypercapnic ventilatory response to passive head‐up tilt have been reported previously (Hazlett & Edgell, 2018; Richardson et al., 2002; Wang et al., 2004; Yoshizaki et al., 1998), there are fewer data available for head‐down tilt.

Recently Murray et al. (2021), demonstrated that 4 h of 6° head‐down bed rest enhanced rather than diminished the hypercapnic ventilatory response, which they attributed to central chemoreflex stimulation due to cephalic CO_2_ accumulation. They also observed no influence of head‐down bed rest on the cardiorespiratory responses to hypoxia. These data contrast with longer duration bed rest studies (20 days) which found lower CO_2_ chemosensitivity (Katayama et al., 2004) and an attenuated hypoxic ventilatory response after 16 days of exposure to microgravity (Prisk et al., 2000). Skow et al. (2014) reported that orthostatic stress induced by 45° and 90° HDT had no effect on central respiratory chemoreflex loop gain while Tymko et al. (2015) subsequently showed that cerebrovascular CO_2_ reactivity, a potentially important mechanism affecting central chemoreflex gain (Carr et al., 2021), remained well‐regulated in both the middle and posterior cerebral artery during 45° and 90° HDT.

It is important to recognise, however, that Skow et al. (2014) and Tymko et al. (2015) used hyperoxic rebreathing to diminish the peripheral chemoreflex and reduce the arterial, venous and brain tissue $P_{CO_{2}}$ gradients (Ainslie & Duffin, 2009; Fan et al., 2010) while Murray et al. (2021) used a steady state technique (5% inspired CO_2_).

Understanding the physiological effects of postural change on the respiratory system is important as such changes in physiological function may have implications not only in microgravity, but also for patient care such as when employing the steep Trendelenburg position during surgery, anaesthesia and shock, and during sleep. Based on the conflicting evidence in the literature, the aim of the current study was therefore to undertake a comprehensive assessment of the acute effects of steep head‐down tilt (50°) on the ventilatory response elicited via central and peripheral chemoreflex stimulation by exposure to steady state normoxia, normoxic hypercapnia (central and peripheral chemoreflex), hyperoxic hypercapnia (central chemoreflex) and hypoxia (peripheral chemoreflex). The study also investigated the role of cerebral haemodynamics in the ventilatory responses during head‐down tilting by measuring middle cerebral artery blood flow velocity (MCAv) as an index of cerebral blood flow, using transcranial Doppler ultrasound. We hypothesised that steep HDT would augment the steady state hypercapnic ventilatory response.

## 2 METHODS

### 2.1 Ethical approval

Healthy subjects with no history of neuromuscular, cardiorespiratory or cerebrovascular disease were recruited to participate in this study. Ethical approval was obtained from the King's College London Ethics Committee (RESCM‐19/20‐8487), and research was conducted in accordance with the *Declaration of Helsinki*. Written informed consent was obtained prior to the start of the study, and subjects were made aware of their right to withdraw from the study at any point.

### 2.2 Equipment and participant set‐up

A manually operated tilt table (King's College London, UK) was used in protocol 1, which allowed both head‐up and head‐down tilts, while an electrically operated tilt table (Plinth2000 Ltd, Stowmarket, UK) was used in protocol 2, which allowed only head‐down tilt from the supine posture to be performed. In both protocols, a corrugated foam overlay was placed on the table to prevent subjects from sliding during tilting, and an additional strap situated across the ankles was used to secure subjects during tilting. All subjects in both protocols were familiarised with head‐up and head‐down tilt prior to commencing data collection. With the subject wearing a noseclip, respiratory flow was measured via a mouthpiece, using a pneumotachograph (4800 series, Hans Rudolph Inc., Shawnee, KS, USA) and associated differential pressure transducer (Spirometer, ADInstruments, Oxford, UK). The distal end of the pneumotachograph was attached to a two‐way non‐rebreathing valve (2700 series, Hans Rudolph Inc.). An open circuit was used to deliver a continuous supply of medical air to the inspiratory port of the two‐way non‐rebreathing valve via a low volume (2.5 L) reservoir bag. Respired gases were continuously analysed using a fast‐gas analyser (ML 206, ADInstruments), which was connected to a side port on the pneumotachograph via a fine‐bore catheter. The inspired gas could be enriched with 100% nitrogen, 100% Oxygen or 100% carbon dioxide from cylinders (BOC, Guildford, UK). Manual titration was performed to achieve the required inspired gas concentration. Four inspired gas conditions were used: normoxia (21% O_2_, balance N_2_), poikilocapnic hypoxia (12% O_2_, balance N_2_), normoxic hypercapnia (21% O_2_, 5% CO_2_, balance N_2_) and hyperoxic hypercapnia (95% O_2_, 5% CO_2_). A pulse oximeter (Sat 805 pulse oximeter, Charter Kontron, Milton Keynes, UK) attached to the subject's finger was used to monitor oxygen saturation ($S_{pO_{2}}$). Minute ventilation (${\dot{V}}_{E}$) was calculated as a product of tidal volume (*V*_t_) and respiratory rate (RR).

Beat‐by‐beat arterial blood pressure was measured continuously using finger photoplethysmography (Finapres, Ohmeda 2300, BOC Healthcare, Inglewood, CO, USA). Diastolic and systolic blood pressure were measured directly from the recording while mean arterial pressure (MAP) was calculated using the weighted mean calculation (⅓ systolic + ⅔ diastolic) values. Heart rate (HR) was measured using electrocardiogram (ECG) electrodes positioned in the lead II configuration (ML132 bioamplifier, ADInstruments). In protocol 2, a 2 MHz transcranial Doppler ultrasound (TCD) system (Doppler Box Compumedics, Singen, Germany) was used to measure right mean MCAv. The ultrasound probe was positioned over the right transtemporal window above the zygomatic ridge between the lateral canthus of the eye and the auricle pinna and was held in place by an adjustable headset to ensure signal quality was controlled and consistent. Cerebrovascular conductance (CVC) was calculated by dividing MCAv by MAP (Rimoy et al., 1991). To aid interpretation of cerebrovascular measurements we calculated a proxy for cerebrovascular reactivity to CO_2_ from the slope of the change in MCAv and $P_{\mathrm{ETC}O_{2}}$ in normoxia and hypercapnia when supine and during HDT.

All data were recorded and displayed in real time using LabChart software (Chart version 8, ADInstrument), with an analog to digital conversion rate of 1 kHz (Powerlab 16, ADInstruments).

### 2.3 Protocol

#### 2.3.1 Protocol 1

After securing the subjects to the tilt table, the study commenced with a 5‐min period of breathing in a 60° head‐up posture, after which the tilt table was rotated to the 50° HDT position for a further 10 min. At the end of the 10 min tilt, the table was returned to the 0° supine position and following a short rest period of approximately 10 min, during which all cardiorespiratory variables returned to baseline, the protocol was repeated under a different gas condition. Four steady gas conditions were employed, normoxia, poikilocapnic hypoxia, normoxic hypercapnia (central and peripheral chemoreflex) and hyperoxic hypercapnia (central chemoreflex only). Subjects were exposed to the different test gases throughout the entire protocol (baseline and HDT). Gases were applied in random order and subjects were unaware of the gas condition during each trial.

#### 2.3.2 Protocol 2

A second study was undertaken on a separate occasion to confirm the results from protocol 1 and to examine the role of cerebral blood flow in the ventilatory responses to 50° HDT. Measurements of MCAv were performed continuously under each gas condition. Unlike protocol 1, after securing the subjects to the tilt table, the study commenced with a 5‐min period of breathing in the supine 0° position after which the tilt table was rotated to 50° HDT for a further 10 min. At the end of the 10 min tilt, the table was returned to the 0° position and after a short rest period of approximately 10 min during which all cardiorespiratory variables returned to baseline, the protocol was repeated for the other gas condition. To make the protocol less burdensome for participants, only two gas conditions were employed, normoxia and normoxic hypercapnia.

### 2.4 Statistical analysis

Data are expressed as mean (standard deviation, SD). Mean breath‐by‐breath and beat‐by‐beat values for the ventilatory, cardiovascular and cerebral artery blood flow velocity data, respectively, were calculated for the final minute of the baseline and for each minute of the 50° head‐down tilt.

#### 2.4.1 Protocol 1

To examine the effects of HDT under different gas conditions, data were grouped according to time and gas condition and a two‐way repeated measures ANOVA with Greenhouse–Geisser correction performed. Post‐hoc testing to examine the effect of head‐down tilt was performed using Dunnett's correction and significance levels determined by comparing each minute of HDT to the baseline 60° head‐up tilt posture.

#### 2.4.2 Protocol 2

To examine the effects of HDT under different gas conditions, a two‐way repeated measures mixed effects analysis with Greenhouse–Geisser correction was employed as data for all variables were unavailable in one subject in minutes 9 and 10 of the HDT with normoxia. Post‐hoc testing to examine the effect of head‐down tilt was performed using Dunnett's correction and determined significance levels by comparing each minute of HDT to the supine baseline posture. Student's paired *t*‐test was used to examine the effects of HDT on cerebrovascular reactivity. Statistical significance was accepted with a *P* < 0.05.

## 3 RESULTS

### 3.1 Protocol 1. 60° head up to 50° head down

**FIGURE 1:**
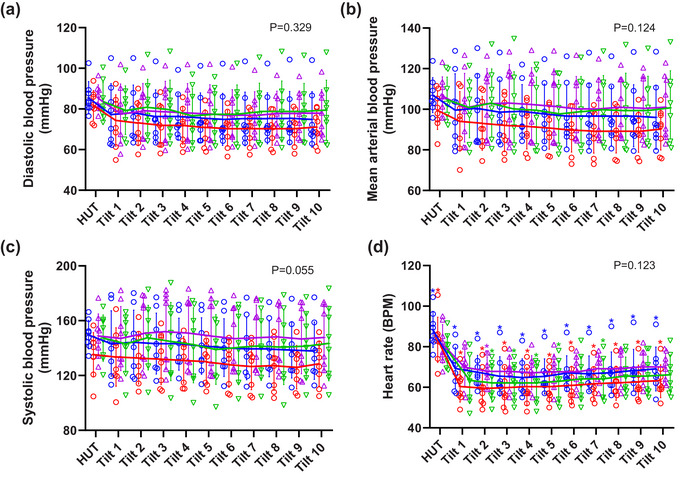
Protocol 1. Cardiovascular responses to HDT during normoxia (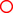), hypoxia (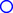), normoxic hypercapnia (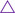), and hyperoxic hypercapnia (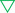) (*n* = 8): (a) diastolic blood pressure, (b) mean arterial blood pressure, (c) systolic blood pressure, and (d) heart rate. Individual data with solid lines representing mean values and vertical bars representing SD. X axis represents time of tilt in minutes. HUT, head up tilt position at baseline. ^*^Significant difference compared to baseline 60° HUT posture (see Tables 1 and 2 for individual *P*‐values). The numerical *P*‐value represents the main effect (ANOVA) of the gas condition. All data *n* = 8.

Eight healthy subjects (mean [SD] age 26 [10] years, two female, BMI 25.7 [3.49] kg/m^2^) were studied. Overall, there were no statistically significant main effects of gas condition on diastolic, systolic, MAP and heart rate (Table 1, Figure 1a–d). 50° HDT resulted in significant reductions in both diastolic pressure (*P* = 0.019) and heart rate (*P* = 0.001) with significant interaction between HDT and gas condition for heart rate (*P* = 0.015). When compared to baseline, post‐hoc testing showed significant reductions in heart rate (Figure 1d, Table 1) were observed throughout the HDT during normoxia (*P *< 0.01) and hypoxia (*P *< 0.05) and at minute (min) 2 during normoxic hypercapnia (*P* = 0.0488) and at min 2–5 and min 7 (*P *< 0.05) during hyperoxic hypercapnia. Significant reductions in diastolic pressure during the HDT (Figure 1a, Table 2) were also observed during normoxia (min 5–10, *P *< 0.05) and hypoxia (min 5–6, *P *< 0.05).

**TABLE 1:** *P*‐values when comparing heart rate at baseline to each minute of HDT during protocol 1.

Time	*P*‐value for change in heart rate from baseline (60° head‐up position)			
Tilt	Normoxia	Hypoxia	Normoxic hypercapnia	Hyperoxic hypercapnia
HUT vs. Tilt minute 1	0.006	0.021	0.187	0.090
HUT vs. Tilt minute 2	0.005	0.006	0.049	0.028
HUT vs. Tilt minute 3	0.006	0.004	0.090	0.023
HUT vs. Tilt minute 4	0.004	0.007	0.100	0.028
HUT vs. Tilt minute 5	0.005	0.003	0.092	0.033
HUT vs. Tilt minute 6	0.007	0.005	0.071	0.066
HUT vs. Tilt minute 7	0.005	0.006	0.131	0.039
HUT vs. Tilt minute 8	0.008	0.014	0.155	0.054
HUT vs. Tilt minute 9	0.008	0.018	0.215	0.057
HUT vs. Tilt minute 10	0.011	0.029	0.134	0.070

HDT, head‐down tilt; HUT, head‐up tilt.

**TABLE 2:** *P*‐values when comparing diastolic blood pressure at baseline to each minute of HDT during protocol 1.

Time	*P*‐value for change in diastolic blood pressure from baseline (60° head‐up position)			
Tilt	Normoxia	Hypoxia	Normoxic hypercapnia	Hyperoxic hypercapnia
HUT vs. Tilt 1	0.450	0.277	0.370	0.890
HUT vs. Tilt 2	0.231	0.198	0.434	0.985
HUT vs. Tilt 3	0.111	0.074	0.508	0.868
HUT vs. Tilt 4	0.057	0.059	0.428	0.417
HUT vs. Tilt 5	0.024	0.043	0.401	0.579
HUT vs. Tilt 6	0.033	0.050	0.244	0.732
HUT vs. Tilt 7	0.026	0.061	0.182	0.785
HUT vs. Tilt 8	0.022	0.057	0.221	0.871
HUT vs. Tilt 9	0.035	0.081	0.248	0.822
HUT vs. Tilt 10	0.042	0.091	0.297	0.927

HDT, head‐down tilt; HUT, head‐up tilt.

**FIGURE 2:**
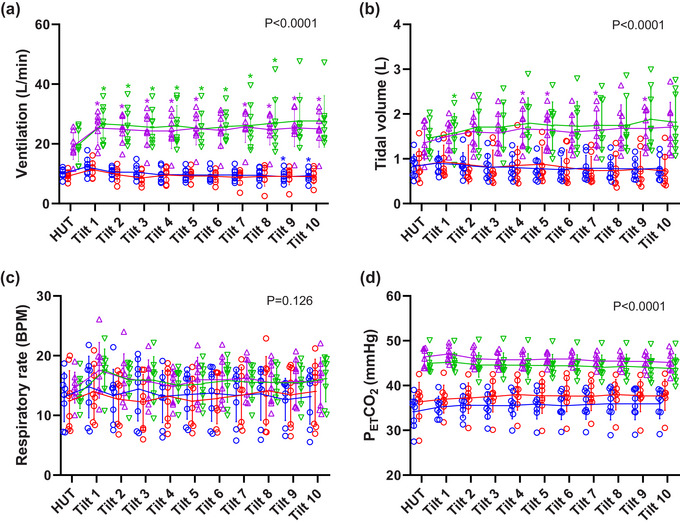
Protocol 1. Respiratory responses to HDT during normoxia (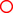), hypoxia (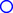), normoxic hypercapnia (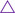), and hyperoxic hypercapnia (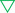) (*n* = 8): (a) ventilation, (b) tidal volume, (c) respiratory rate, and (d) $P_{\mathrm{ETC}O_{2}}$. Individual data with solid lines representing mean values and vertical bars representing SD. X axis represents time of tilt in minutes. HUT, head up tilt position at baseline. *Significant difference compared to baseline 60° HUT posture (see Table 3 for individual *P*‐values). The numerical *P*‐value represents the main effect (ANOVA) of gas condition. All data *n* = 8.

Statistically significant main effects of gas condition were observed for ventilation (*P *< 0.0001), tidal volume (*P *< 0.0001) and $P_{\mathrm{ETC}O_{2}}$ (*P *< 0.0001) with elevated levels under normoxic and hyperoxic hypercapnia compared to normoxia and hypoxia (Figure 2a, b, d). No significant effects were observed in respiratory rate between any gas condition. 50° HDT resulted in a significant main effect on ventilation (*P* = 0.001) and significant interactions between HDT and gas condition for ventilation (*P* = 0.004) and tidal volume (*P* = 0.011).

Post‐hoc testing demonstrated that despite there being no change in $P_{\mathrm{ETC}O_{2}}$ (Figure 2d), compared to baseline (60° head‐up position), ventilation increased significantly during 50° HDT during both normoxic (Figure 2a, all *P *< 0.05 except min 6, Table 3) and hyperoxic hypercapnia (Figure 2a, all *P *< 0.05 except min 8–10). The changes in ventilation during normoxic and hyperoxic hypercapnia appeared primarily to be due to changes in tidal volume (Figure 2b, normoxic hypercapnia min 4, *P* = 0.011, min 5, *P* = 0.022, min 7, *P* = 0.015, and min 9, *P* = 0.040, hyperoxic hypercapnia min 1, *P* = 0.049). No changes in ventilation were seen under normoxic or hypoxic conditions (Figure 2a) at any time during HDT, except for a small but significant reduction in ventilation at min 9 and 10 during hypoxia (min 9, *P* = 0.034, min 10, *P* = 0.025).

**TABLE 3:** *P*‐values when comparing ventilation at baseline to each minute of HDT during protocol 1.

Time	*P*‐value for change in ventilation from baseline (60° head‐up position)			
Tilt	Normoxia	Hypoxia	Normoxic hypercapnia	Hyperoxic hypercapnia
HUT vs. Tilt 1	0.150	0.096	**0.011**	**0.004**
HUT vs. Tilt 2	0.974	0.137	**0.002**	**0.003**
HUT vs. Tilt 3	0.650	0.742	**0.007**	**0.011**
HUT vs. Tilt 4	>0.999	0.984	**0.030**	**0.015**
HUT vs. Tilt 5	>0.999	0.680	**0.022**	**0.031**
HUT vs. Tilt 6	0.996	0.950	0.081	**0.021**
HUT vs. Tilt 7	0.965	0.611	**0.011**	**0.043**
HUT vs. Tilt 8	>0.999	0.861	**0.016**	0.057
HUT vs. Tilt 9	0.992	**0.034**	**0.049**	0.074
HUT vs. Tilt 10	0.9987	**0.025**	**0.026**	0.078

Values in bold indicate statistical significance. HDT, head‐down tilt; HUT, head‐up tilt.

### 3.2 Protocol 2. Supine to 50° head down

Ten healthy young subjects (mean [SD] age 27 [11] years, four female, body mass index [BMI] 23.3 [1.7] kg/m^2^) were studied. One subject was common to both protocols. All data *n* = 10 except for normoxia, min 9 and 10 during HDT where *n* = 9.

**FIGURE 3:**
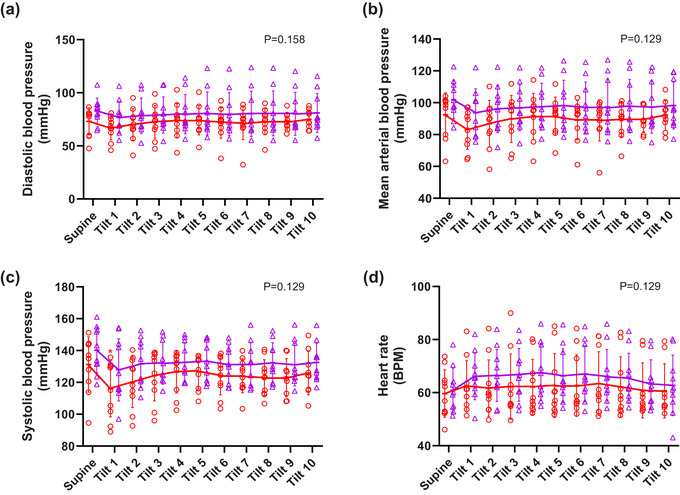
Protocol 2. Cardiovascular responses to HDT during normoxia (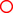) and normoxic hypercapnia (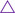) (*n* = 10, except normoxia min 9 and 10 when *n* = 9). (a) diastolic blood pressure, (b) mean arterial blood pressure, (c) systolic blood pressure, and (d) heart rate. Individual data with solid lines representing mean values and vertical bars representing SD. X axis represents time of tilt in minutes and supine position at baseline. *Significant difference compared to baseline supine posture. The numerical *P*‐value represents the main effect (ANOVA) of gas condition. All data *n* = 10 except for normoxia min 9 and 10 during HDT where *n* = 9.

Similar to protocol 1, overall, there were no statistically significant main effects of gas condition on diastolic, systolic, MAP and heart rate (Figure 3a–d). 50° HDT resulted in significant reductions in systolic pressure (*P* = 0.012) with no significant interaction between HDT and gas condition for any variable. Post‐hoc testing showed that other than a brief fall in systolic blood pressure in minute 1 during the tilt in normoxia (Figure 3c, *P* = 0.043), 50° HDT from a supine baseline position had no effect on cardiovascular parameters.

Similar to protocol 1, statistically significant main effects of gas condition were observed for ventilation (*P* = 0.0008), tidal volume (*P* = 0.008) and $P_{\mathrm{ETC}O_{2}}$ (*P *< 0.0001) with elevated levels during hypercapnia.

50° HDT had significant main effects on ventilation (*P* = 0.048) and respiratory rate (*P* = 0.036) with significant interactions between HDT and gas condition for ventilation (*P* = 0.005) and tidal volume (*P* = 0.038).

**FIGURE 4:**
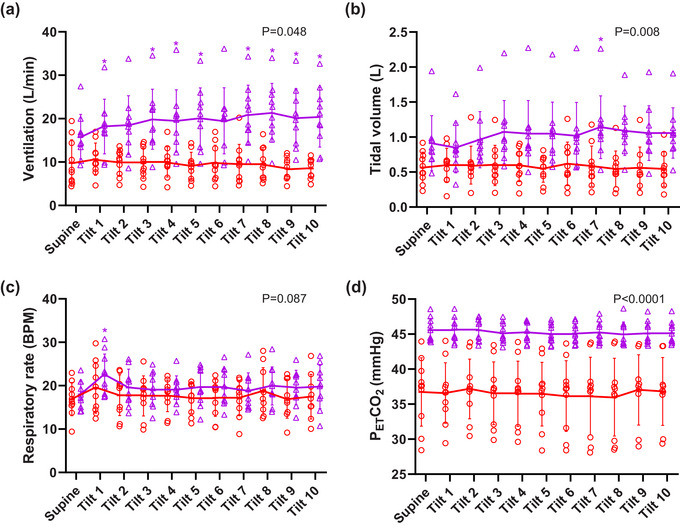
Protocol 2. Respiratory responses to HDT during normoxia (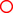) and normoxic hypercapnia (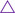) (*n* = 10, except normoxia min 9 and 10 when *n* = 9) for (a) ventilation, (b) tidal volume, (c) respiratory rate, and (d) $P_{\mathrm{ETC}O_{2}}$. Individual data with solid lines representing mean values and vertical bars representing SD. X axis represents time of tilt in minutes and supine position at baseline.*Significant difference compared to baseline supine posture. The numerical *P*‐value represents the main effect (ANOVA) of gas condition. All data *n* = 10 except for normoxia min 9 and 10 during HDT where *n* = 9.

As previously observed in protocol 1, despite there being no change in $P_{\mathrm{ETC}O_{2}}$ (Figure 4d) post‐hoc testing showed significant increases in ventilation during hypercapnic 50° HDT only (all *P *< 0.05 except min 2 and 6, Figure 4a, Table 4). No change was seen during normoxia (Figure 4a, Table 2). As in protocol 1, the changes in ventilation appeared primarily to be due to changes in tidal volume (Figure 4b, min 7 *P* = 0.0013), although a statistically significant increase in respiratory rate was observed at min 1 during HDT in hypercapnia (Figure 4c, *P* = 0.0196).

**TABLE 4:** *P*‐values when comparing ventilation at baseline to each minute of HDT during protocol 2.

Time	*P*‐value for change in ventilation from baseline (supine position)	
Tilt	Normoxia	Hypercapnia
HUT vs. Tilt 1	0.962	**0.027**
HUT vs. Tilt 2	>0.999	0.053
HUT vs. Tilt 3	>0.999	**0.026**
HUT vs. Tilt 4	>0.9999	**0.040**
HUT vs. Tilt 5	0.998	**0.040**
HUT vs. Tilt 6	>0.999	0.105
HUT vs. Tilt 7	>0.999	**0.017**
HUT vs. Tilt 8	>0.999	**0.007**
HUT vs. Tilt 9	0.788	**0.016**
HUT vs. Tilt 10	0.8631	**0.023**

All data *n* = 10 except for the normoxia condition min 9 and 10 during HDT where *n* = 9. Values in bold indicate statistical significance. HDT, head‐down tilt; HUT, head‐up tilt.

**FIGURE 5:**
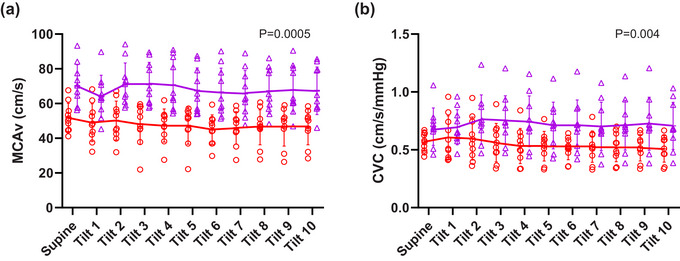
Protocol 2. Cerebrovascular responses to HDT in normoxia (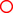) and normoxic hypercapnia (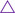): (a) MCAv and (b) CVC during the 10 min HDT compared to baseline supine posture (all *n* = 10, except normoxia minute 9 and 10 when *n* = 9). Individual data with solid lines representing mean values and vertical bars representing SD. X axis represents time of tilt in minutes and supine position at baseline. The numerical *P*‐value represents the main effect (ANOVA) of gas condition. All data *n* = 10 except for normoxia min 9 and 10 during HDT where *n* = 9.

**FIGURE 6:**
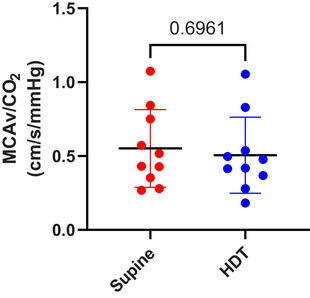
Protocol 2. Cerebrovascular reactivity calculated while supine (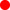) and during HDT (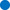) (*n* = 10). Bars represent mean (SD). *P*‐value represents results of *t*‐test.

Statistically significant main effects of gas condition were observed for MCAv (*P* = 0.0005) and CVC (*P* = 0.004) with elevated levels under hypercapnic conditions (Figure 5). There were, however, no significant main effects of HDT or interaction between gas condition and HDT on MCAv or CVC. There was no difference in cerebrovascular reactivity to CO_2_ between supine and head‐down conditions (Figure 6).

## 4 DISCUSSION

These data demonstrate that acute 50° head‐down tilt augments the ventilatory response to steady state normoxic and hyperoxic hypercapnia. There was no change in ventilation during HDT under normoxia or steady state poikilocapnic hypoxia, or any difference in the augmented ventilatory response to normoxic compared to hyperoxic hypercapnia.

While the effects of head‐up tilt on ventilatory response to inhaled gases have been studied previously (Hazlett & Edgell, 2018; Richardson et al., 2002; Wang et al., 2004; Yoshizaki et al., 1998), the acute ventilatory responses to HDT have not been explored to the same degree. No marked differences in the hypercapnic and hypoxic ventilatory responses have been found between seated and supine postures (Mannix et al., 1984; Rigg et al., 1974; Slutsky et al., 1980; Weissman et al., 1982; Xie et al., 1991) using either steady state or rebreathing methods. Similarly, using hyperoxic rebreathing to diminish the peripheral chemoreflex and reduce the arterial to tissue to venous $P_{CO_{2}}$ gradients (mixing gain) (Ainslie & Duffin, 2009; Carr et al., 2023; Duffin, 2011), Skow et al. (2014) found no difference in ventilatory responsiveness to increased CO_2_ across five body positions ranging from 90° head‐up tilt (HUT) to 90° HDT. Conversely, Murray et al. (2021) reported that, although there was no effect on the hypoxic ventilatory response, 4 h of 6° head‐down bed rest enhanced the ventilatory response to steady‐state hypercapnia (5% CO_2_). As they observed no changes in MCAv following HDT they hypothesised that the change in ventilatory response was due to cephalic CO_2_ accumulation from cerebral congestion, although the exact mechanism remained unclear. The results of the current study using steady‐state normoxic and hyperoxic hypercapnia of the same magnitude (5% CO_2_) during steep HDT are consistent with those from Murray et al.

Khoo et al. (1982) originally proposed the engineering model describing the feedback loops associated with chemoreflex control of breathing with loop gain, the overall sensitivity of the system being the product of controller, mixing/feedback and plant gains. Controller gain describes the strength of the controller response in relation to a deviation between the input and output signal and reflects the respiratory output for a given level of chemoreceptor stimulation. It has been suggested that the controller gain can be modulated by cortical input (Hayashi & Sinclair, 1991) and feedback from lung stretch receptors (Moreira et al., 2007), baroreceptors (McMullan et al., 2009) and peripheral chemoreceptors (Blain et al., 2010). The lack of change in ventilation during HDT in normoxia and hypoxia, and no difference in the response to normoxic and hyperoxic hypercapnia would suggest cortical input and baroreceptor and peripheral chemoreceptor feedback are not involved. In addition, as an augmented ventilatory response (increased loop gain) was only observed during HDT in normoxic and hyperoxic hypercapnia, during which there was no change in $P_{\mathrm{ETC}O_{2}}$, these data argue against any mechanism that involves altered mixing/feedback gain, such as changes in cardiac output, or plant gain, which includes effects of lung mechanics, respiratory muscle function, or lung ventilation perfusion and shunt. While 30° and 60° HDT has been shown to result in increased pulmonary blood flow heterogeneity (Henderson et al., 2006; Olfert & Prisk, 2004), there are few data available describing the effects of HDT on the arterial partial pressure of CO_2_ ($P_{\mathrm{aC}O_{2}}$) to $P_{\mathrm{ETC}O_{2}}$ gradient. Kalmar et al. (2010) showed a 3 mmHg increase in the $P_{\mathrm{aC}O_{2}}$ to $P_{\mathrm{ETC}O_{2}}$ gradient following 120 min in the steep Trendelenburg position (40°) in patients under general anaesthesia with CO_2_ pneumoperitoneum. Although no changes in $P_{\mathrm{ETC}O_{2}}$ from baseline throughout HDT in normoxic and hyperoxic hypercapnia were observed in the current study, it is possible that an increased $P_{\mathrm{aC}O_{2}}$ to $P_{\mathrm{ETC}O_{2}}$ gradient may explain the augmented ventilation observed. While we cannot discount such changes during HDT, it is important to acknowledge, however, that no change in ventilation was seen during HDT in normoxia and hypoxia when similar changes in $P_{\mathrm{aC}O_{2}}$ to $P_{\mathrm{ETC}O_{2}}$ gradient may have been expected to occur. The data from Kalmar et al. were obtained in mechanically ventilated patients under general anaesthesia with CO_2_ pneumoperitoneum, and unlike the current study, they observed increases in $P_{\mathrm{ETC}O_{2}}$ during HDT.

The lack of change in MCAv and CVC during hypercapnic HDT would also suggest that the mechanism underlying the augmented ventilation was independent of cerebral blood flow (mixing gain). Although our calculation was only a proxy, we also observed no change in CVR with HDT, which is consistent with the results of Tymko et al. (2015) who showed that cerebrovascular CO_2_ reactivity in the anterior and posterior cerebral circulations was unchanged with 45° and 90° HDT. Brain stem CO_2_/[H^+^] is considered the stimulus for the central chemoreceptors (CCRs) (Wang et al., 1993), and is determined by the metabolic rate of brain tissue and its washout by the effects of cerebral blood flow (CBF). The arterial–tissue CO_2_ gradient is directed from brain tissue into the blood, and the accumulation of CO_2_ in brain tissue stimulates CCRs. Increases in $P_{\mathrm{aC}O_{2}}$ decrease the arterial–tissue CO_2_ gradient between the blood and brain tissue, resulting in a reduced washout of CO_2_ from the brain and increased CCR stimulation. $P_{\mathrm{aC}O_{2}}$ is also a modulating factor for cerebral blood flow (Ogoh & Ainslie, 2009) with a higher $P_{\mathrm{aC}O_{2}}$ increasing CBF, which has the effect of increasing the arterial–tissue CO_2_ gradient by increasing the washout of CO_2_ from brain tissue and, therefore, reducing CCR stimulation and attenuating the ventilatory response. The resulting hypercapnic ventilatory response is therefore the balance of these two interacting factors and acts to maintain central [H^+^] (Ainslie & Duffin, 2009; Xie et al., 2006). Control of CBF involves several other interacting regulatory mechanisms including metabolic, autoregulatory, neural and chemical, and endothelial mechanisms (Ainslie & Duffin, 2009). While the cerebrovasculature responds to increased sympathetic activity (Jordan et al., 2000) and CO_2_‐induced increases in CBF are attenuated by elevated sympathetic tone (Jordan et al., 2000), the significant falls in heart rate observed during HDT in protocol 1 would, however, imply reduced sympathetic outflow and argue against a significant role of the sympathetic nervous system in the augmented hypercapnic ventilatory response seen in the current study.

The caudal fluid shifts induced by 50° HDT will result in increased intracranial pressure (ICP) (Kato et al., 2022). Increases in ICP during HDT are likely a result of increased cerebral venous pressure due to gravity‐induced increases in hydrostatic pressure. ICP during HDT may be further enhanced by the increase in cerebral venous blood volume, particularly during steep tilts, which may act to further reduce intracranial compartment compliance (Kato et al., 2022; Kramer et al., 2017). HDT will also result in reduced venous drainage from the head. Marshall‐Goebel et al. (2016) demonstrated a decrease in internal carotid and vertebral artery blood flow alongside increased cross‐sectional area and decreased internal jugular vein blood flow during progressive levels of HDT. Whittle and Diaz‐Artiles (2023) observed no change in common carotid artery cross‐sectional area, but substantial increases in internal jugular vein cross‐sectional area and venous pressure during acute 45° HDT. As the brain is within a closed compartment, alterations in the route of venous outflow and increases in cerebral venous pressure could act to modulate CO_2_‐induced cerebral vasodilatation during hypercapnic HDT potentially resulting in alterations in CCR stimulation and augmented ventilation.

### 4.1 Limitations

Although hypoxia and hypercapnia both act to increase cerebral blood flow and maintain cerebral O_2_ delivery, we did not observe any changes in ventilation during HDT in hypoxia. These findings are in agreement with those obtained by Murray et al. (2021) following 6° head‐down bed rest. An absence of response may have been due to the relatively modest level of hypoxia employed, being insufficient to cause significant alterations in CBF. While reductions in $P_{aO_{2}}$ below ∼50 mmHg cause significant elevations in CBF (Carr et al., 2021), the mean $P_{\mathrm{ET}O_{2}}$ in the current study was 53.6 mmHg. Alterations in $P_{aO_{2}}$ in the range of 60–150 mmHg have little influence on CBF under normal physiological conditions and constant $P_{\mathrm{aC}O_{2}}$ (Brugniaux et al., 2007). The lack of difference in the magnitude of augmented ventilation between normoxic and hyperoxic hypercapnic conditions in protocol 1 would also argue against a significant role for the peripheral chemoreflex in the augmented ventilatory responses.

In the current study, we used transcranial Doppler ultrasound as a measure of cerebral blood flow. Increases in $P_{\mathrm{aC}O_{2}}$ induce arteriolar vasodilatation downstream from conduit vessels, increasing cerebral blood velocity (Willie et al., 2014). Measurements of MCAv represent blood flow only if the diameter of the artery remains constant. The MCA dilates during modest hypercapnia (Coverdale et al., 2014; Valdueza et al., 1997, 1999) such that measures of MCAv underestimate true cerebral blood flow by as much as 20% (Coverdale et al., 2014). In our study, increases in cerebral blood flow during hypercapnia were therefore likely to be larger than the observed increase in MCAv. Measurement of MCAv is commonly used as an index of global cerebral blood flow (Willie et al., 2011). It is important to acknowledge, however, that the cerebral blood flow response shows marked heterogeneity when challenged with a range of physiological stressors (Sato et al., 2012; Skow et al., 2013; Smith et al., 2012; Willie et al., 2012). Measurement of cerebral blood flow velocity in the posterior circulation may therefore have allowed a better understanding of the current results and potential mechanisms.

Although augmented ventilatory responses during hypercapnic HDT were seen in both protocols 1 and 2, slight differences in the time course and magnitude of both the ventilatory and cardiovascular responses occurred between protocols. Ventilation increased immediately at the start of hypercapnic HDT in protocol 1, while the increase in ventilation occurred over a longer time course in protocol 2. Similarly, marked reductions in diastolic BP and significant reductions in heart rate for all tilts were observed in protocol 1, which were not seen in protocol 2. We attribute the differences in cardiorespiratory responses to the different starting postures adopted in each protocol, with head‐down tilts in protocol 1 starting from a 60° head‐up posture therefore inducing greater initial cardiovascular stress. The results from protocol 1 indicated augmented ventilation during HDT in both normoxic and hyperoxic hypercapnia. Hyperoxic hypercapnia was employed in protocol 1 to isolate any effect of the peripheral chemoreceptors. As there was no difference in the magnitude of response between normoxic and hyperoxic hypercapnia, we opted to streamline protocol 2 to make it less burdensome on the participants and therefore employed only normoxic hypercapnia when investigating the effects of HDT on MCAv.

In neither protocol did we observe a significant increase in MAP. During HDT, gravity‐dependent shifts in blood volume would result in increased venous return to the thorax, which would increase cardiac preload, potentially increasing MAP via increases in cardiac output, induced by the Starling mechanism. Such increases in blood volume in the thorax would, however, activate aortic and carotid baroreceptors and cardiopulmonary receptors resulting in reflex bradycardia to counteract the increase in BP. The significant decreases in heart rate observed in protocol 1 reflect arterial baroreflex activation and potentially explain the lack of change in MAP. Similar to our results, Bundgaard‐Nielsen et al. (2009) observed no change in MAP during 45° HDT compared to supine baseline. We attribute the differences in cardiovascular responses between protocols 1 and 2 to the different starting postures and a greater initial cardiovascular stress in protocol 1 (baseline 60° head up).

### 4.2 Implications

The current results are clinically relevant due to the use of CO_2_ insufflation during laparoscopic and robotic surgery in a steep head down Trendelenburg position (30−40°) and resulting in increased levels of $P_{\mathrm{aC}O_{2}}$ (Kalmar et al., 2010). Although permissive mild hypercapnia is frequently employed during laparoscopic surgery, no studies have previously examined the combined effects of hypercapnia and cephalad fluid shifts on respiratory control. The findings are also relevant to microgravity environments such as those experienced on the International Space Station where individuals are exposed to both fluid shifts and elevated atmospheric CO_2_ levels.

### 4.3 Conclusions

We examined the acute effects of steep head‐down tilt (50°) on the ventilatory response elicited via central and peripheral chemoreflex stimulation by exposure to steady‐state normoxia, normoxic hypercapnia, hypoxia and hyperoxic hypercapnia. We observed an augmented ventilatory response during HDT under normoxic and hyperoxic hypercapnia only. The absence of any change in ventilation during exposure to the other gas conditions would suggest the mechanism underpinning these changes was independent of the peripheral chemoreflex, lung mechanics, respiratory muscle function, or change in lung ventilation and perfusion. We observed no significant change in MCAv using transcranial Doppler ultrasound during HDT. We postulate, therefore, that the augmented ventilatory response during steep HDT may involve mechanisms related to the effects of HDT on cerebral venous pressure and venous outflow.

## AUTHOR CONTRIBUTIONS

All work was undertaken in the Human Physiology Laboratories, Centre for Human & Applied Physiological Sciences, Faculty of Life Sciences and Medicine, King's College London, UK. Gerrard F. Rafferty conceived and designed the experiments. Abdulaziz Alsharifi, Gerrard F. Rafferty, Niamh Carter, Akbar Irampaye, and Charlotte Stevens performed the experiments. Gerrard F. Rafferty and Elisa Mejia analysed the data. Gerrard F. Rafferty interpreted the data. Gerrard F. Rafferty, Joerg Steier, and Abdulaziz Alsharifi wrote the manuscript. Gerrard F. Rafferty and Joerg Steier revised the article for important intellectual content. All authors have read and approved the final version of this manuscript and agree to be accountable for all aspects of the work in ensuring that questions related to the accuracy or integrity of any part of the work are appropriately investigated and resolved. All persons designated as authors qualify for authorship, and all those who qualify for authorship are listed.

## CONFLICT OF INTEREST

None declared.

## FUNDING INFORMATION

None.

## Data Availability

The data are available from the corresponding author upon reasonable request.
